# Draft Genome Sequence of *Lactobacillus rhamnosus* CLS17

**DOI:** 10.1128/genomeA.00478-15

**Published:** 2015-05-14

**Authors:** Samat S. Kozhakhmetov, Almagul R. Kushugulova, Saule A. Saduakhasova, Gulnara S. Shakhabayeva, Zhanagul R. Khassenbekova, Askhat B. Molkenov, Ulykbek E. Kairov, Raushan B. Issayeva, Talgat S. Nurgozhin, Zhaxybay S. Zhumadilov

**Affiliations:** a Department of Translational Medicine, Longevity and Global Health, PE Center for Life Sciences, Nazarbayev University, Astana, Kazakhstan; b Department of Organization and Development of Genomic and Personalized Medicine, PE Center for Life Sciences, Nazarbayev University, Astana, Kazakhstan

## Abstract

We announce the draft genome sequence of the type strain *Lactobacillus rhamnosus* CLS17 (2,889,314 nt, with a GC content of 46.8%), which is one of the most prevalent lactic acid bacteria present during the manufacturing process of dairy products; the genome consists of 71 large contigs (>100 bp in size). It contains 2,643 protein-coding sequences, single predicted copies of the 5S, 16S, and 23S rRNA genes, and 51 predicted tRNAs.

## GENOME ANNOUNCEMENT

The human gut microbiome is an organ that provides primary barrier protection against foreign agents. Most of the microorganisms are different strains of commensal bacteria that are colonized in the gut. Gut flora influence food metabolism and have an antagonistic effect on different pathogens and immunomodulatory properties (1). One of the main species of gut flora is in the genus *Lactobacillus*.

*Lactobacillus* is the largest genus in the family *Lactobacillaceae* and belongs to the phylum *Firmicutes*, in the human microbiome. *Lactobacillus* sp. is a key member of the human microflora (2). They colonize the gastrointestinal and urogenital tracts, the oral cavity, and breast milk. This genus is a symbiont to humans and has a high probiotic characteristic (3).

The strain was grown under microaerobic conditions in MRS broth (Fluka, Buchs, Switzerland) at 37°C. Genomic DNA extraction was performed with a Wizard Genomic DNA purification kit (Promega, Madison, WI, USA).

For genome sequencing, we used Roche 454 GS (FLX Titanium) pyrosequencing (4). All of the reads were assembled using Newbler Assembler version 2.3 (454 Life Science), which generated 71 large contigs (>100 nt). The total genome assembly of 2,889,314 nt (23.9-fold coverage of the genome) and GC content (46.8%) were in good correlation with published genome sequences of other strains of this species (46.3 to 46.9%). There are single predicted copies of the 5S, 16S, and 23S rRNA genes and 51 predicted tRNAs.

Polymorphisms in genes are responsible for the adaptive properties of the strain (transport of sugar and metabolism, heat shock proteins, regulators of intracellular pH, alkaline-shock proteins, bile salt, heat shock proteins, and heavy metal adhesins) identified.

Polymorphisms in the gene-encoding proteins related to oxidative stress, such as catalase, thiol peroxidase, glutathione peroxidase, halo peroxidase, four thioredoxins, four glutathione reductases, five NADH-oxidases, two NADH peroxidases, peptide methionine sulfoxide reductases, and alkyl hydroperoxide reductase, as well as intracellular accumulation of Mn^2+^ ions, were identified.

### Nucleotide sequence accession numbers. 

The genome sequence of *Lactobacillus rhamnosus* CLS17 available in GenBank under the accession numbers JYCS01000001 to JYCS01000071.
